# Finite Element-Based Biomechanical Evaluation of Patient-Specific Insoles for a Pediatric Patient with Hereditary Spastic Paraplegia Using the Taguchi Method

**DOI:** 10.3390/bioengineering12121323

**Published:** 2025-12-04

**Authors:** Dhifaf Muhi Alsaleh, Fuat Bilgili, Meral Bayraktar, Yunus Ziya Arslan

**Affiliations:** 1 Department of Mechanical Engineering, Faculty of Mechanical Engineering, Yildiz Technical University, Istanbul 34349, Türkiye; mbarut@yildiz.edu.tr; 2 Department of Orthopedics and Traumatology, Istanbul Faculty of Medicine, Istanbul University, Istanbul 34093, Türkiye; 3 Department of Robotics and Intelligent Systems, Institute of Graduate Studies in Science and Engineering, Turkish-German University, Istanbul 34820, Türkiye; yunus.arslan@tau.edu.tr

**Keywords:** pes planovalgus, hereditary spastic paraparesis, Taguchi method, ANOVA, Minitab, finite element analysis, patient specific foot insole

## Abstract

Customized foot orthoses are widely used to manage plantar pressure and improve structural support in children with hereditary spastic paraparesis. However, the combined biomechanical effects of insole design parameters remain insufficiently quantified. This study employed a patient-specific three-dimensional finite element model to evaluate the influence of four design factors (arch height, heel cup depth, insole thickness, and material type, namely ethylene-vinyl acetate [EVA], thermoplastic polyurethane [TPU], and rubber) on four biomechanical metrics: plantar pressure distribution, von Mises stress, strain, and total deformation. Nine orthotic configurations, defined by a Taguchi L9 orthogonal array, were simulated under a vertical ground reaction force equal to 1.1× body weight. The configuration with an arch height of 42 mm, heel cup depth of 20 mm, thickness of 10 mm, and EVA material achieved the lowest peak plantar pressure (0.087 MPa). Arch height was the dominant factor for plantar pressure (79.4% of variance), deformation (68.1%), and strain (48.2%), while heel cup depth was most influential for stress (40.2%). Material type contributed minimally to plantar pressure and deformation but had a greater effect on stress (11.6%) and strain (15.0%). Thickness played a secondary role, particularly in deformation (19.9%) and strain (22.3%). These findings demonstrate the feasibility of using finite element modeling combined with the Taguchi method to systematically evaluate and optimize orthotic design parameters. Specifically, the study demonstrates that optimized personalized insoles can substantially reduce peak plantar pressure and improve load distribution in a pediatric patient with HSP, pes planovalgus, and flexed-knee gait, providing a potentially effective noninvasive intervention to prevent secondary complications and improve gait mechanics.

## 1. Introduction

Hereditary spastic paraparesis (HSP) encompasses a clinically and genetically heterogeneous group of inherited neurodegenerative disorders [1]. The main clinical symptom is progressive lower limb spasticity, often accompanied by foot deformities, and typically more pronounced than associated muscle weakness [2]. This progressive spasticity substantially impairs gait, reduces functional independence, and deteriorates overall quality of life in affected individuals [3,4,5].

Although pes planovalgus (PPV) is not considered a primary clinical manifestation of HSP, it may develop as a secondary deformity resulting from chronic lower-limb spasticity, neuromuscular imbalance, and compensatory adaptations associated with the progressive nature of the disorder. The medial, longitudinal, and transverse arches of the foot are essential for maintaining biomechanical integrity and functional locomotion. PPV is a progressive deformity characterized by collapse of the medial longitudinal arch, medial displacement of the talus, and forefoot abduction [6]. It is typically classified as either flexible flattening under weight-bearing or rigid, with arch collapse present in both loaded and unloaded conditions [7,8,9]. PPV has a profound impact on ankle joint kinematics during gait, frequently resulting in compromised push-off mechanics and altered force transmission across the lower limb. This dysfunction often leads to a cascade of compensatory biomechanical changes, including flexed-knee gait, tibial and femoral rotational deviations, anterior pelvic tilt, and secondary spinal malalignments [10,11]. During the stance phase of gait, approximately 55% of the plantar pressure is transmitted through the hindfoot, particularly the talus and calcaneus underscoring the importance of targeted support in orthotic intervention [12].

The foot and ankle architecture confers both stability and elasticity, enabling efficient weight-bearing, shock absorption, and propulsion during gait. Foot deformities are among the most common musculoskeletal disorders, frequently resulting in pain, functional limitations, and compromised quality of life [13,14]. Among various conservative treatments, insoles represent a widely prescribed non-invasive intervention, aiming to restore biomechanical alignment, redistribute plantar loads, and alleviate symptoms [15,16]. Compared to prefabricated devices, custom-made insoles have demonstrated superior outcomes in correcting flexible deformities and enhancing comfort, owing to their tailored fitting and capacity to accommodate individual anatomical variations [17]. Insoles incorporating medial arch support have shown measurable improvements in foot alignment, particularly in pediatric populations with flatfoot deformities [18,19,20,21]. A variety of polymeric materials including microcellular rubber, ethylene vinyl acetate (EVA), silicone, thermoplastic polyurethane (TPU), and polyurethane are routinely employed in insole fabrication due to their favorable cushioning, durability, and shock-absorbing properties [22,23,24,25].

To evaluate the mechanical efficacy of foot orthoses, a range of experimental and computational approaches has been developed to quantify plantar pressure and shear stress distributions during gait [26,27,28,29]. Among these, finite element (FE) modeling of the foot–ankle complex has emerged as a powerful tool for simulating internal load transfer and assessing the biomechanical performance of orthotic interventions [30,31,32,33]. Complementary investigations using cadaveric specimens and in vivo pressure measurement techniques have further advanced the understanding of plantar load dynamics and tissue response [34,35].

This study aims to identify the optimal insole design for a patient diagnosed with HSP, presenting with concurrent PPV, flexed-knee gait, and hallux valgus. To date, no studies have applied a Taguchi method combined with finite element modeling to optimize insole design for patients with this combination of neuromuscular and biomechanical impairments. A series of insole configurations was evaluated using an FE modeling approach to simulate plantar pressure distribution under physiological loading. A Taguchi orthogonal array design was employed to systematically assess the effects of key design parameters, with analysis of variance (ANOVA) used to identify the most influential factors. This methodology provides a structured framework for personalized insole optimization, addressing a clear gap in the literature and offering potential for improving biomechanical performance and functional outcomes in pediatric patients with complex foot deformities.

## 2. Materials and Methods

### 2.1. Demographic Information and Biomechanical Measurements of the Participant

Ethical approval for this study was granted by the Biruni University Scientific Research Ethics Committee (Approval Number: 2024-BİAEK/06-46 on 20 January 2025).

**Figure 1:**
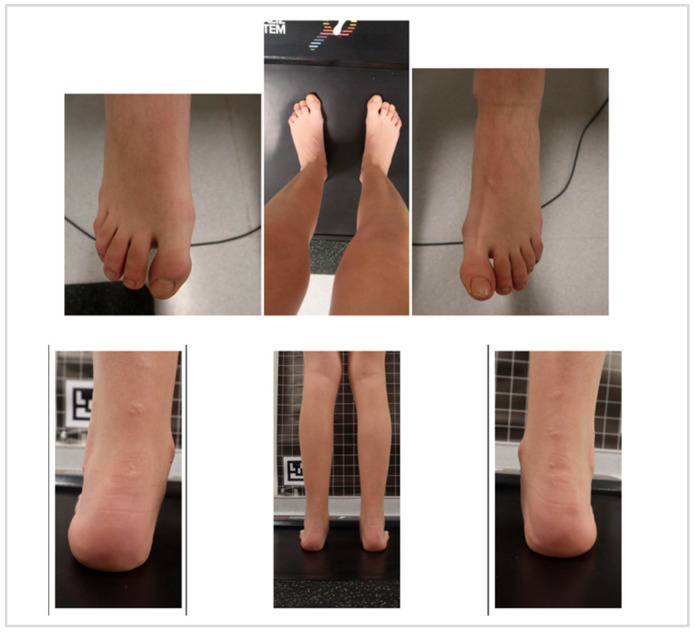
The patient with Hereditary spastic paraparesis (HSP), pes planovalgus (PPV), flexed-knee gait, and hallux valgus defect.

In this study, a subject-specific FE model of foot–insole–ground interaction was developed to assess the biomechanical performance of customized orthotic insoles for a pediatric patient diagnosed with HSP, presenting with PPV, flexed-knee gait, and hallux valgus (Figure 1).

**Figure 2:**
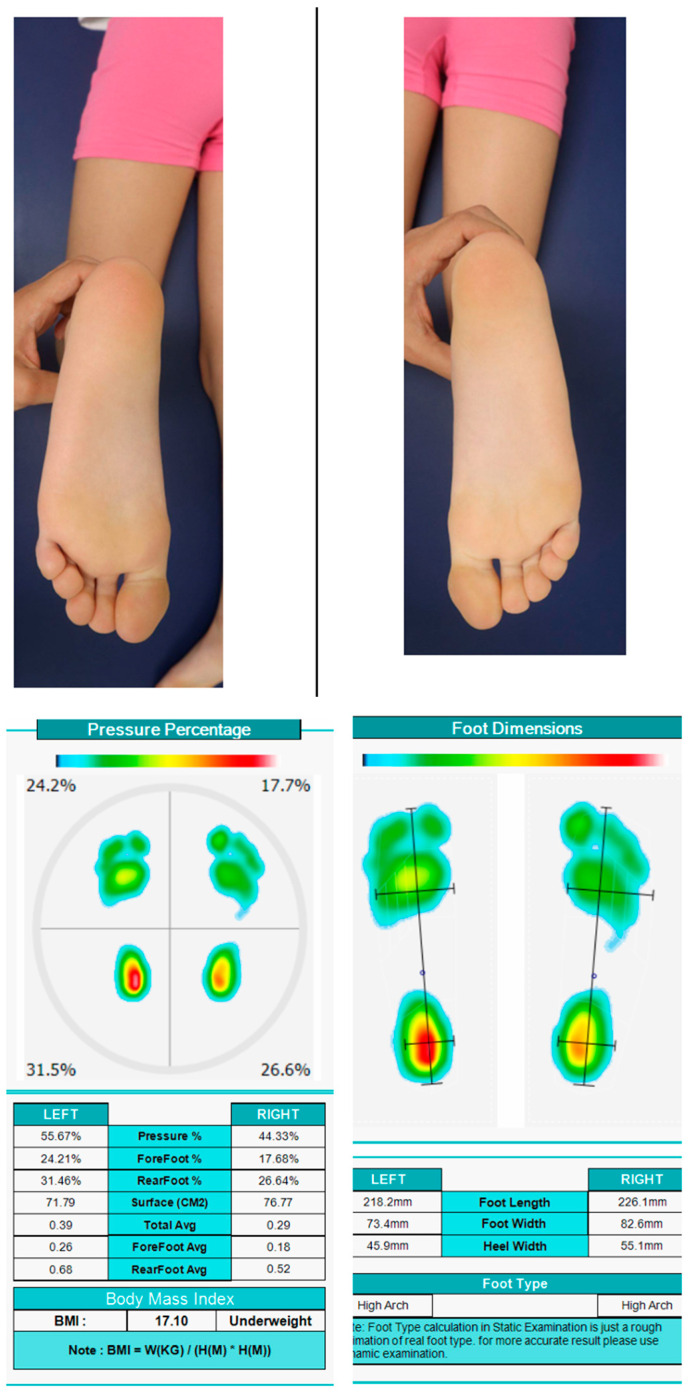
Pressure percentage and feet dimensions of patient.

The study included a pediatric female diagnosed with HSP at 11 years with a body mass of 39 kg, presenting with PPV, flexed-knee gait, and hallux valgus. She has received regular physiotherapy and previous interventions including lower limb surgeries and botulinum toxin injections. The patient consistently uses AFOs and night immobilizers. Clinical gait and postural assessments showed persistent sagittal-plane deviations and mild postural asymmetries, confirming suitability for subject-specific FE modeling and personalized insole evaluation. Figure 2 shows pressure percentage and feet dimensions of patient.

To evaluate abnormal plantar pressure, plantar pressure data were collected using a pedobarography system (Analysis System 50 × 45 cm, Novel GmbH, Munich, Germany), which is equipped with a dense array of capacitive pressure sensors to provide high-resolution measurements during the stance phase of gait.

**Figure 3:**
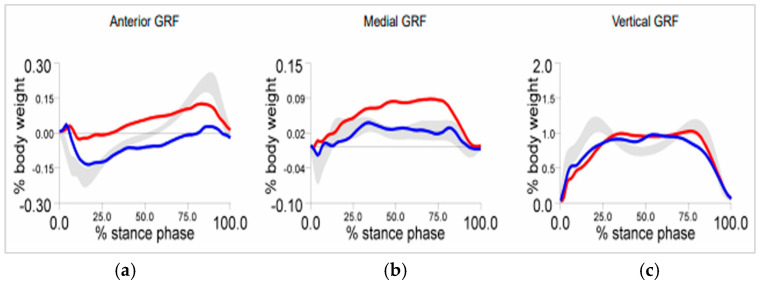
(**a**) Anterior, (**b**) medial, and (**c**) vertical components of the experimentally obtained ground reaction force (GRF). Grey area: reference range; blue curve: left foot; red curve: right foot.

The vertical, anterior, and medial components of the ground reaction force were obtained during the patient’s walking through gait analysis performed at Biruni University Hospital using the Bertec force plate system (Bertec Corporation, Columbus, OH, USA), which incorporates strain gauge load cells for precise measurement of ground reaction forces and moments (Figure 3). The vertical ground reaction force was recorded as 421.8 N, corresponding to 1.1 times the patient’s body weight, and this value was applied at the ground–insole interface to simulate weight-bearing during the midstance phase.

### 2.2. The Foot and Insole Modeling

The geometric model of the foot was reconstructed using surface scan data obtained via the COMB application (Custom Orthotic Modeling and Building, Version 08; Comb O&P, LLC; Chardon, OH, USA), ensuring anatomically accurate representation. The scanned geometry model was then processed in Autodesk Meshmixer (version 3.5, Autodesk Inc., San Rafael, CA, USA) for cleanup and anatomical refinement, and SOLIDWORKS 2020 (Dassault Systèmes, Vélizy-Villacoublay, France) was used to design the customized insole configurations.

### 2.3. Subject-Specific Finite Element Model Construction

The FE analysis encompassed nine custom-designed insole configurations, systematically constructed by varying four critical design parameters: medial arch height, heel cup depth, insole thickness, and material type. The materials selected included commonly used orthotic polymers, namely EVA, TPU, and rubber. Each configuration was evaluated for four key biomechanical metrics: plantar pressure distribution, von Mises stress, strain, and total deformation.

To optimize the experimental design and reduce the number of simulation trials without compromising result validity, a Taguchi L9 orthogonal array was employed [36]. This statistical framework facilitated efficient exploration of the design space while capturing the main effects of the selected parameters. To further assess the relative influence and statistical significance of each design factor on the biomechanical metrics, analysis of variance (ANOVA) was performed using Minitab^®^ Statistical Software, version 21.4 (Minitab LLC, State College, PA, USA).

FE simulations were performed in ANSYS Workbench (version 2024 R2, ANSYS Inc., Canonsburg, PA, USA). To account for geometric nonlinearity, the Large Deflection option was activated in the analysis settings. The contact between the plantar surface and the insole was defined as frictional, using a pure penalty formulation with a friction coefficient of 0.5 [37], allowing realistic sliding and separation during loading. These nonlinear settings ensured accurate representation of the foot insole interaction. The insole materials (EVA, TPU, and Rubber) were modeled as linear elastic isotropic solids, with their mechanical properties listed in Table 1.

**Figure 4:**
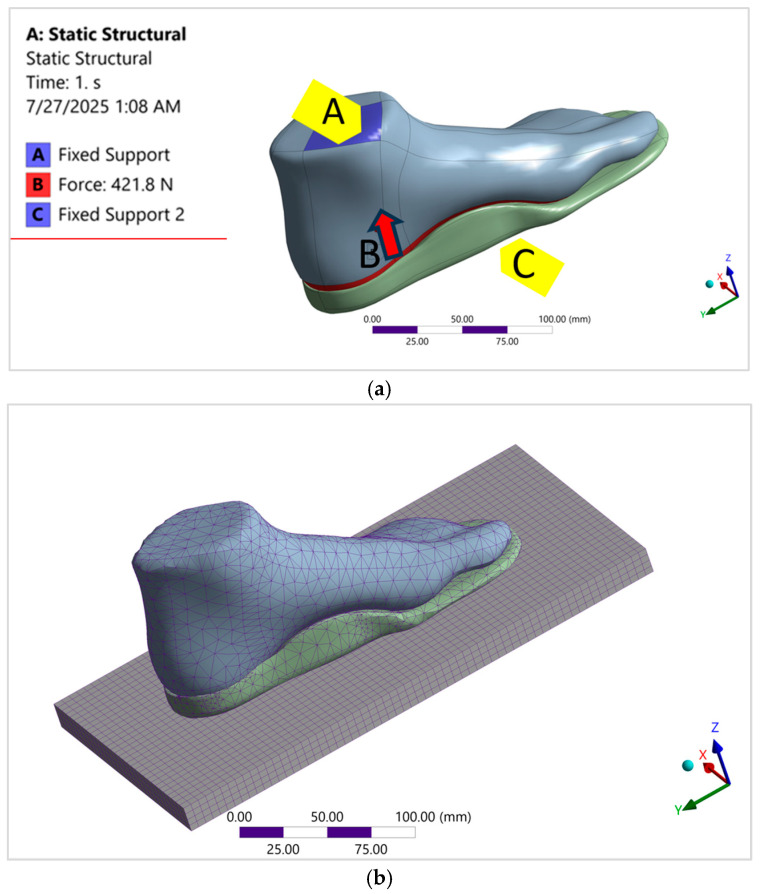
(**a**) The 3D foot model was constrained at anatomically appropriate fixation points and loaded with biomechanically representative forces to simulate physiological conditions and (**b**) a three-dimensional foot geometry was meshed with unstructured tetrahedral elements to ensure accurate representation of anatomical complexity.

To replicate realistic loading conditions during walking, boundary conditions were applied to represent the midstance phase of the gait cycle. The proximal surfaces of the tibia, fibula, and encapsulated soft tissue were fully constrained to reflect anatomical fixation. A rigid ground plate was coupled beneath the insole to control degrees of freedom and apply external loading, as shown in Figure 4a. A mesh sensitivity analysis was performed (details are provided in Section 2.5. Mesh Sensitivity Analysis), and a 5 mm mesh size with tetrahedral elements was selected to improve resolution for the simulations (Figure 4b).

**Figure 5:**
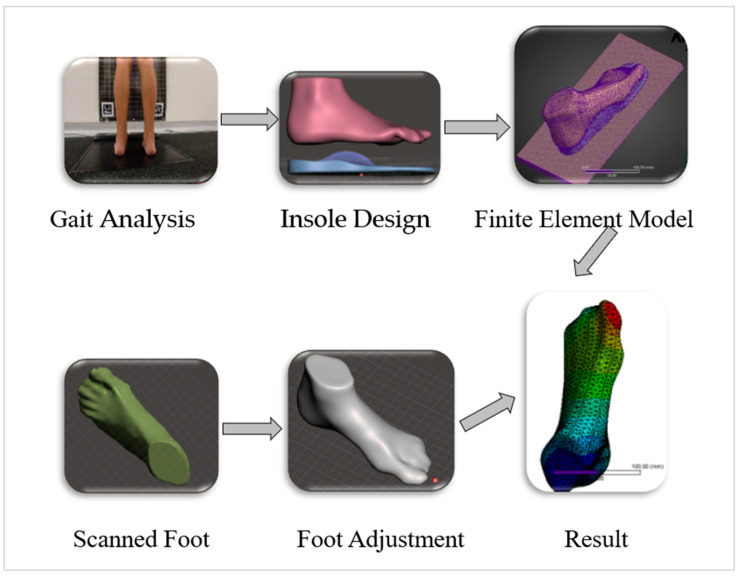
The integrated modeling framework to analyze the mechanical response of the foot–insole system under static loading condition.

This integrated modeling framework was employed to analyze the mechanical response of the foot–insole system under static loading condition, generating biomechanical outputs for each insole to support the design optimization. A schematic representation of the modeling workflow, including foot geometry acquisition, insole design variations, and FE simulation setup is shown in Figure 5.

Von Mises stress, equivalent strain, and total deformation were evaluated over the plantar surface of the foot model in contact with the insole, and the peak values were extracted to quantify the maximum mechanical response under each orthotic configuration.

### 2.4. Computational Evaluation of Foot Orthoses Design Parameters Using Taguchi Method

A series of nine customized foot orthosis configurations were developed based on three-dimensional (3D) surface scans obtained under minimal weight-bearing conditions (<5% body weight), thereby preserving the natural arch contour. Each insole was analyzed systematically varying four design parameters.

**Figure 6:**
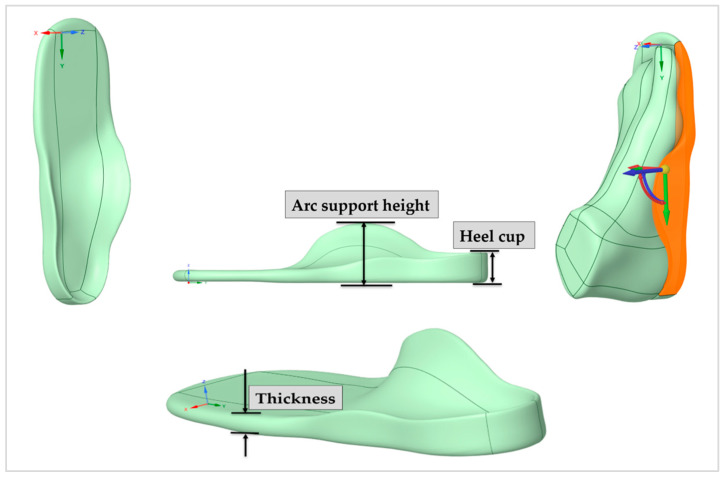
The foot orthosis design parameters, including heel cup, arch support height, and thickness.

Figure 6, each at three levels: arch height (30, 37, and 42 mm), heel cup depth (16, 18, and 20 mm), insole thickness (8, 10, and 12 mm), and material type (EVA, TPU, and rubber) (Table 2).

The parameter levels selected for arch height, heel cup depth, and insole thickness were determined based on a combination of patient-specific foot morphology and clinically established orthotic design guidelines. The arch height values (30, 37, and 42 mm) were chosen to span the corrective range commonly used in pediatric flatfoot and valgus deformity management, ensuring both anatomical accommodation and medial arch support, as supported by prior FE modeling studies [30,36,41]. Heel cup depths of 16, 18, and 20 mm reflect the recommended 14–20 mm range for calcaneal stabilization in children, allowing appropriate rearfoot alignment while maintaining wearer comfort. Insole thickness levels (8, 10, and 12 mm) were selected to represent the standard manufacturing limits used in pediatric custom orthoses, balancing cushioning and bulk.

The resulting nine insole designs (D_1_–D_9_) are summarized in Table 3, which outlines the specific factor–level combinations tested in the FE simulations. To optimize the design process, minimize the number of simulation trials without compromising validity, and reduce computational cost while ensuring a comprehensive evaluation, a Taguchi L9 orthogonal array was employed [36]. According to Taguchi methodology, the relative influence and statistical significance of each design factor on the biomechanical metrics were assessed using the “smaller-is-better” criterion for plantar pressure and von Mises stress, and the “larger-is-better” criterion for total deformation and strain. This approach enabled the identification of the optimal factor levels for each outcome measure.

ANOVA was conducted using Minitab^®^ Statistical Software (Version 21.4, Minitab LLC, State College, PA, USA) within the Taguchi design framework to compute the main effects of each design factor and to determine their relative influence on the mechanical outcomes.

### 2.5. Mesh Sensitivity Analysis

A mesh sensitivity analysis was performed using four tetrahedral mesh sizes (8 mm, 7 mm, 5 mm, and 4 mm). These mesh sizes were selected based on previous studies [41,42]. The 4 mm mesh served as the fine-mesh reference. Geometric nonlinearity (Large Deflection) and frictional contact (μ = 0.5) were included to capture realistic deformation and load transfer. Those mesh sizes were simulated in six representative models ($D_{2},$ $D_{4},\;D_{6},\;D_{7},\;D_{8},\;D_{9}$).

The 5 mm mesh produced minimal differences relative to the 4 mm mesh in peak plantar pressure (0.35–3.83%) and total deformation (0.11–2.82%), satisfying the commonly used <5% convergence criterion [43]. The 7 mm mesh retains deviations < 10% for most models, which remains acceptable for nonlinear foot tissue simulations [44], where soft-tissue material behavior and contact interactions introduce inherent numerical variability. In contrast, the 8 mm mesh caused substantial deviations in total deformation (up to 22.75%) and was therefore excluded. Based on these results, the 5 mm tetrahedral mesh was adopted for all analyses as the optimal balance between convergence and computational efficiency. The detailed mesh convergence results are presented in Table A1 in Appendix A.

## 3. Results

**Figure 7:**
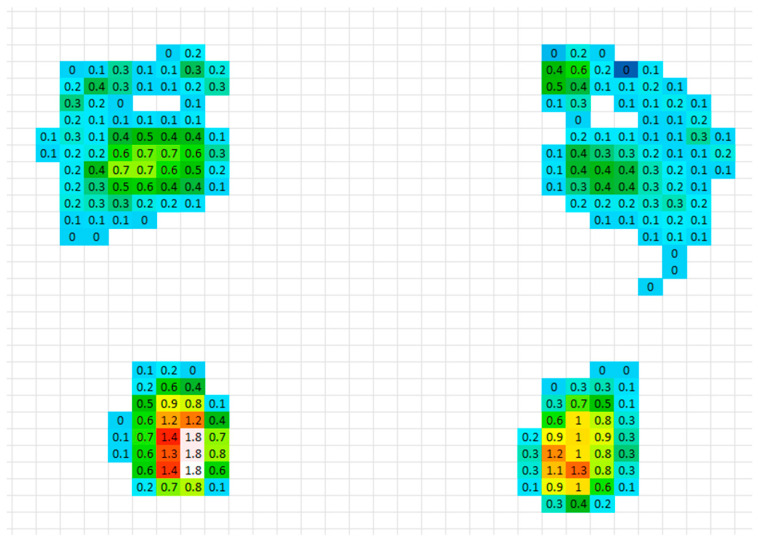
Plantar pressure map of the patient in barefoot condition.

The measured plantar pressure distribution of the patient under barefoot conditions is presented in Figure 7. The maximum plantar pressure recorded was 0.176 MPa.

**Figure 8:**
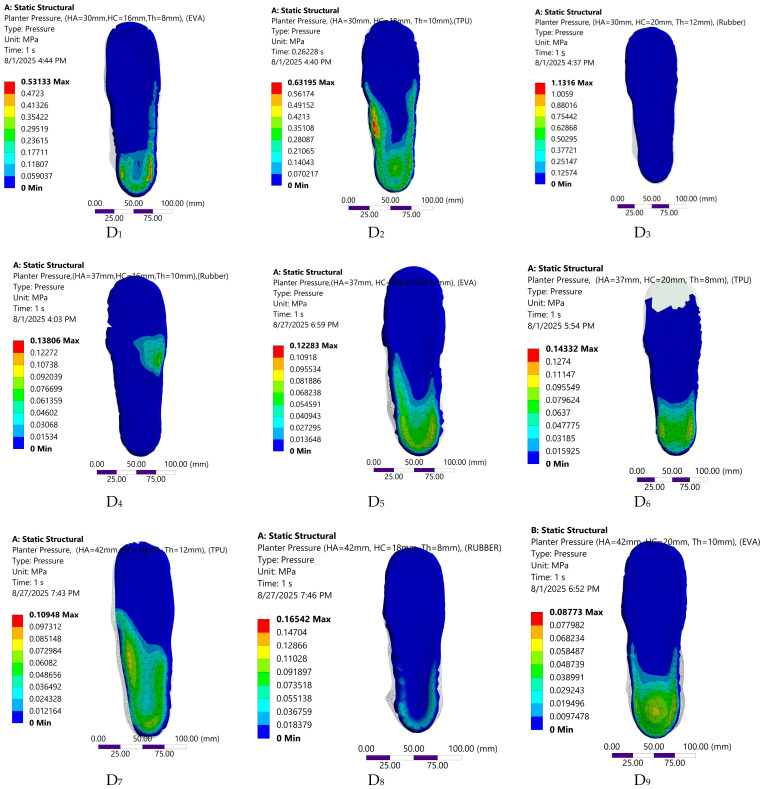
Visual representation of plantar pressure distribution across the nine customized orthotic insole design configurations.

**Figure 9:**
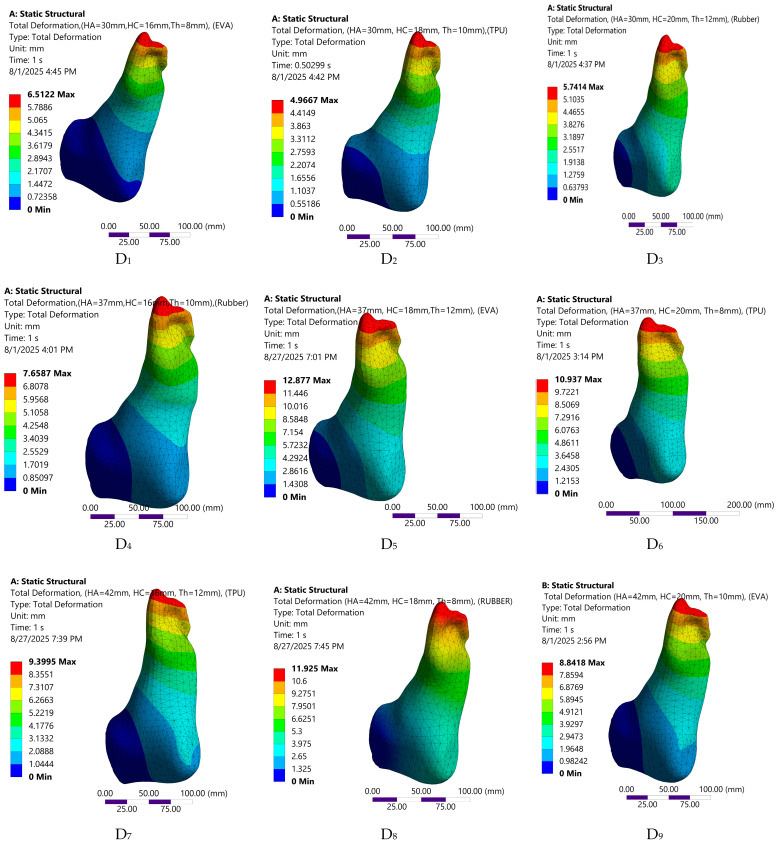
Visual representation of deformation distribution across the nine customized orthotic insole design configurations.

**Figure 10:**
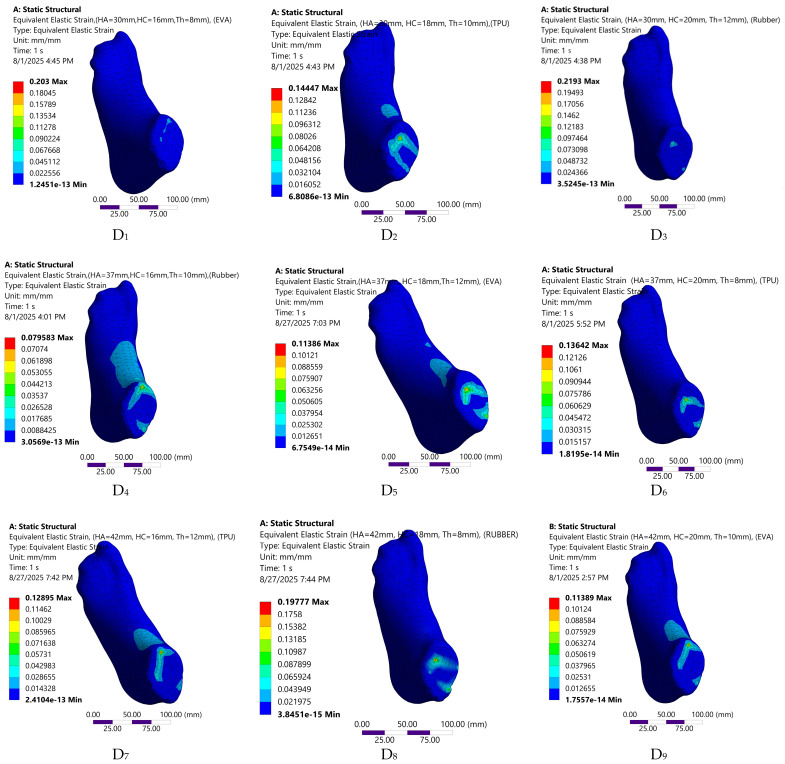
Visual representation of strain distribution across the nine customized orthotic insole design configurations.

**Figure 11:**
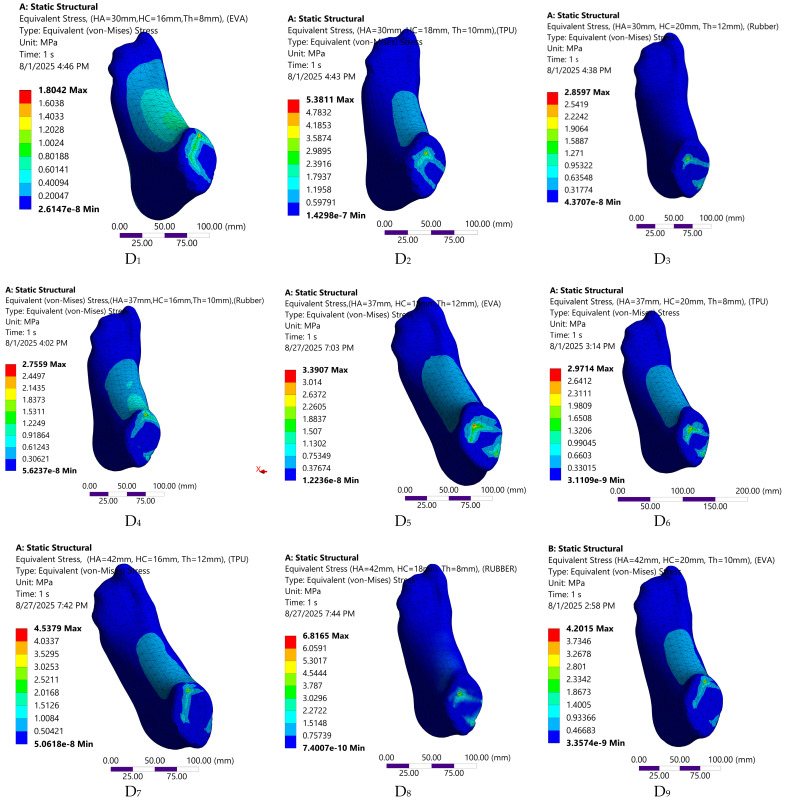
Visual representation of stress distribution across the nine customized orthotic insole design configurations.

The FE predicted biomechanical responses of the nine insole configurations are depicted in Figure 8, Figure 9, Figure 10 and Figure 11. The simulations captured variations in plantar pressure (Figure 8), deformation (Figure 9), strain (Figure 10), and equivalent stress (Figure 11) under identical boundary and loading conditions, enabling the identification of the relative importance of design parameters.

Table 4 summarizes the predicted values for the nine orthotic configurations. Compared with the maximum measured barefoot value in the laboratory (0.176 MPa, Figure 7), all insole configurations, except $D_{3}$, exhibited lower peak plantar pressures (Table 5). The configuration with an arch height of 42 mm, a heel cup of 20mm, a thickness of 10 mm, and EVA material (D_9_) achieved the lowest peak plantar pressure (0.087 MPa).

**Figure 12:**
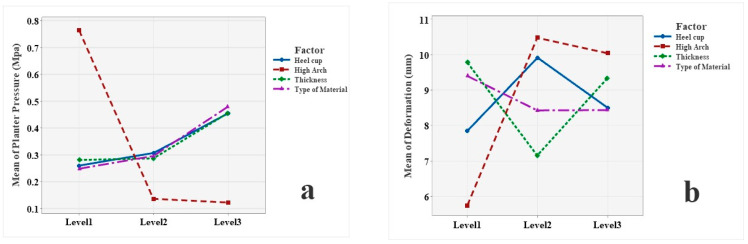

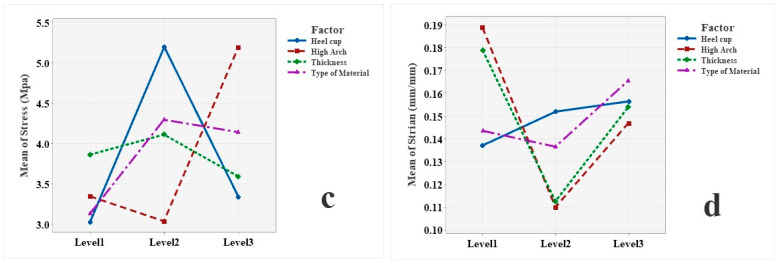
Mean effect of the four design factors at each level on the (**a**) plantar pressure, (**b**) deformation, (**c**) stress, and (**d**) strain.

The mean effect of each design factor (arch support height, heel cup height, insole thickness, and material type) across its three levels is illustrated in Figure 12. Increasing arch support height substantially reduced plantar pressure (Figure 12a). However, higher arch supports were associated with increased deformation (Figure 12b) and elevated stress (Figure 12c), suggesting a redistribution of loading rather than a uniform reduction.

Heel cup depth exhibited mixed effects across the responses. Deeper heel cups moderately increased deformation (Figure 12b) and stress (Figure 12c), while their effect on plantar pressure (Figure 12a) and strain (Figure 12d) remained limited.

Insole thickness played a secondary but relevant role. Greater thickness reduced strain (Figure 12d) and contributed to a moderate increase in plantar pressure (Figure 12a). However, excessive thickness increased deformation (Figure 12b), likely due to greater compressibility under loading.

Material type had a modest influence on plantar pressure (Figure 12a) and deformation (Figure 12b). Among the tested materials, TPU (11 MPa) demonstrated intermediate mechanical behavior, whereas the softer rubber (6 MPa) exhibited greater mean strain values but comparable stress to TPU. This pattern indicates that deformation behavior was influenced primarily by geometry rather than stiffness alone. The ANOVA confirmed that arch height and heel cup depth were the dominant factors affecting stress (39.3% and 40.2% of variance, respectively), while arch height and thickness governed strain (48.2% and 22.3%, respectively). The more compliant rubber material allowed localized deformations under identical boundary conditions, leading to slightly elevated strain levels in some configurations. In contrast, the highly compliant EVA (1.66 MPa) absorbed more energy and distributed load more uniformly, thereby reducing both stress and strain peaks. These findings are consistent with previous finite element studies, which reported that insole geometry and support height have a stronger influence on internal stresses and tissue deformation than material stiffness alone [41,45].

The relative importance of each design factor was quantified through ANOVA, as summarized in Table 5. For instance, the sum of squares associated with arch support height for plantar pressure was 0.8109, accounting for 79.4% of the variation and indicating its dominant role in reducing peak foot pressure. This was followed by material type (8.7%), heel cup depth (6.1%), and insole thickness (5.8%). In the case of deformation, arch height again showed the strongest effect, with a sum of squares of 42.09, contributing 68.1% to the reduction in deformation. Thickness (19.9%) and heel cup depth (11.5%) followed, while material type had a minimal influence (3.6%). For stress, heel cup depth was identified as the most influential factor, with a sum of squares of 8.34 (40.2%), followed by arch height (8.14; 39.3%), material type (2.41; 11.6%), and thickness (0.44; 2.1%). Regarding strain, arch height remained the dominant factor, with a sum of squares of 0.1410 (48.2%), followed by thickness (22.3%), material type (15.0%), and heel cup depth (14.5%).

## 4. Discussion

The use of orthopedic insoles is a widely adopted and effective intervention for managing adolescent flatfoot deformities. However, their therapeutic efficacy is strongly influenced by both geometric configuration and material stiffness. Optimizing the design of personalized insoles has considerable potential to enhance load distribution, improve foot biomechanics, and prevent secondary complications [46,47]. Empirical evidence indicates that customized insoles can reduce peak plantar stress by up to 40% compared with flat-surfaced designs. Furthermore, increasing insole thickness has been shown to promote a more uniform stress distribution and reduce peak stress values by approximately 10% [48,49].

In this study, we employed a subject-specific FE modeling approach, integrated with the Taguchi method and statistical analysis, to optimize the design of customized foot orthoses for a pediatric patient with HSP accompanied by pes planovalgus and flexed-knee gait. The results demonstrated that arch support height was the most influential factor in reducing peak plantar pressure, accounting for 79.4% of the variance according to ANOVA. Higher arch supports (42 mm and 37 mm) effectively redistributed plantar loads, thereby reducing localized pressure concentrations. These findings are consistent with previous reports highlighting the biomechanical benefits of elevating the medial longitudinal arch in managing flexible flatfoot and associated gait impairments [38,50,51,52].

Heel cup depth, on the other hand, had the greatest effect on peak stress 40.2%, underscoring its role in enhancing rearfoot stabilization. Deeper heel cups (20 mm) improve calcaneal control, reducing excessive pronation and improving alignment of the subtalar joint. These findings, aligned with prior experimental and clinical evidence, suggest that rearfoot control is pivotal in orthotic management of neuromuscular foot deformities [53].

Material type with EVA, TPU, and rubber used in the orthoses significantly influenced strain (15.0%) and stress (11.6%) (Table 5).

Softer materials demonstrated superior damping characteristics, thereby lowering localized strain and von Mises stress. The use of softer materials, as corroborated by previous FE simulations and clinical investigations, underscores the critical role of material compliance in enhancing shock attenuation and redistributing plantar pressure [36,54].

Insole thickness influenced plantar pressure 5.8% but contributed less significantly to internal mechanical behavior. This indicates that while thickness may modulate surface pressure through cushioning, its biomechanical role is secondary to shape and material selection.

The optimized insole configuration, featuring EVA material with design parameters arc height = 42 mm, heel cup = 20 mm, and thickness = 10 mm (D_9_), achieved a peak plantar pressure of 0.087 MPa. This represents a 50.15% reduction compared with the barefoot average plantar pressure of 0.176 MPa. Previous FE studies have reported plantar pressure reductions ranging from 9.44% to 16.50% with custom-made insoles of varying stiffness in diabetic neuropathic feet during standing balance conditions [54,55]. Haris et al. (2021) evaluated 3D-printed midsole insoles with honeycomb and gyroid infill patterns (at approximately 20% density) using TPU in healthy participants, reporting a maximum reduction of ~10% in the heel region during walking [56]. Wong et al. (2020) reported up to 37% forefoot pressure reduction with EVA-based orthotic insoles in diabetic patients [57].

This study has several limitations related to methodological choices. A single-subject FE model was employed to reduce computational demand, which limits generalizability but provides consistent trends for evaluating insole design parameters [58,59]. Foot geometry was reconstructed using surface scanning, which captures the external anatomical shape while avoiding the time, cost, and complexity of medical imaging. This approach enables efficient modeling of load-transfer patterns relevant to insole performance without requiring detailed internal tissue data. Static midstance loading was represented using vertical ground reaction force, as it is the dominant and most stable component of foot loading during gait [60,61]. Muscle, tendon, and shear forces were not explicitly modeled; however, their omission does not affect the relative ranking of design factors under identical boundary conditions. Body weight and foot width were not normalized [32,33,34,35,36,41], which may influence absolute plantar pressure values but not the relative improvements observed with optimized insoles. Moreover, this study did not include an experimental validation of the simulated insole configurations. While plantar pressure was experimentally measured under barefoot conditions to provide a reference dataset, in-shoe plantar pressure measurements could not be obtained due to clinical constraints related to the pediatric case. Such measurements would have required specialized equipment and prolonged data acquisition sessions, which were not feasible for the child’s comfort and medical condition in our study.

Several measures were implemented to enhance the numerical reliability of the simulations. These included a mesh convergence analysis, incorporation of nonlinear contact and geometric effects, and the use of material properties obtained from previously validated studies in pediatric foot biomechanics. The findings provide a quantitative basis for selecting orthotic features such as arch height, heel cup depth, and material stiffness, which can guide clinicians and orthotists in designing more effective personalized foot orthoses. Overall, the study serves as a proof of concept for integrating FE-based optimization into pediatric orthotic parameter selection.

## 5. Conclusions

This study demonstrated the applicability of a FE approach combined with the Taguchi method for personalized insole design in a pediatric patient with HSP, presenting with pes planovalgus, flexed-knee gait, and hallux valgus. The results indicated that arch height was the most influential factor for reducing plantar pressure, deformation, and strain, whereas heel cup depth primarily affected stress distribution. Insole thickness and material type, although less dominant, provided additional capacity to refine insole performance. Overall, the findings emphasize the biomechanical importance of geometric parameters (arch and heel support) over material stiffness in redistributing plantar loads in the pediatric case examined. It should be noted, however, that these results are patient-specific and derived from a single-subject finite element model. The identified optimal configuration and dominant factors may vary in individuals with different foot morphologies, walking patterns, or severity of neuromuscular deformity. Future research is intended to expand this work by incorporating multi-subject, anatomically detailed, and muscle-driven simulations, together with normalized anthropometric indices, to enhance generalizability and clinical applicability.

## Figures and Tables

**Table 1:** The material properties of the components in the finite element model.

Material	Elastic Modulus (MPa)	Poisson’s Ratio	References
EVA	1.66	0.49	[38]
TPU	11	0.45	[37]
Rubber	6	0.45	[39]
Foot	50	0.4	[40]
Ground	210,000 (Steel)	0.3	[41]

**Table 2:** Foot orthosis design factors and their levels.

Design Factor	Level1	Level2	Level3
Arc support height (mm)	30	37	42
Heel cup height (mm)	16	18	20
Thickness (mm)	8	10	12
Materials	EVA	TPU	Rubber

**Table 3:** An L9 orthogonal array structured the design.

Trial Number	Code	Design Factor			
		Arch Height (mm)	Heel Cup (mm)	Thickness (mm)	Material Type
1	$D_{1}$	30	16	8	EVA
2	$D_{2}$	30	18	10	TPU
3	$D_{3}$	30	20	12	RUBBER
4	$D_{4}$	37	16	10	RUBBER
5	$D_{5}$	37	18	12	EVA
6	$D_{6}$	37	20	8	TPU
7	$D_{7}$	42	16	12	TPU
8	$D_{8}$	42	18	8	RUBBER
9	$D_{9}$	42	20	10	EVA

**Table 4:** L9 orthogonal array and FE analysis results, showing predicted biomechanical outcomes: peak plantar pressure, total deformation, von Mises stress, and strain.

Trial Number	Code	Plantar Pressure (MPa)	Deformation (mm)	Stress (MPa)	Strain (mm/mm)
1	$D_{1}$	0.53	6.51	1.80	0.20
2	$D_{2}$	0.63	4.96	5.38	0.14
3	$D_{3}$	1.13	5.74	2.85	0.21
4	$D_{4}$	0.13	7.65	2.75	0.07
5	$D_{5}$	0.12	12.87	3.39	0.11
6	$D_{6}$	0.14	10.93	2.97	0.13
7	$D_{7}$	0.10	9.39	4.53	0.12
8	$D_{8}$	0.16	11.92	6.81	0.19
9	$D_{9}$	0.08	8.84	4.20	0.11

**Table 5:** ANOVA results for the predicted peak plantar pressure, deformation, von Mises stress, and strain, all derived from a fractional factorial design incorporating four factors at three levels each.

Design Factor	Sum of Squares for Plantar Pressure, Deformation, Stress and Strain			
	Plantar Pressure	Deformation	Stress	Strain
Arch Height	0.81 (79.4%)	42.09 (68.1%)	8.14 (39.3%)	0.14 (48.2%)
Heel Cup	0.06 (6.1%)	7.10 (11.5%)	8.34 (40.2%)	0.03 (14.5%)
Thickness	0.05 (5.8%)	12.28 (19.9%)	0.44 (2.1%)	0.07 (22.3%)
Type of material	0.08 (8.7%)	2.25 (3.6%)	2.41 (11.6%)	0.06 (15.0%)

## Data Availability

The data supporting the findings of this study are available from the corresponding author upon reasonable request. No publicly archived datasets were generated due to patient privacy considerations.

## Appendix A

**Table A1:** Mesh sensitivity analysis of the foot–insole finite element models.

Models	MS (mm)	NG	NF	NI	EG	EF	EI	PPP(MPa)	% ΔPPP	TD (mm)	% ΔTD
$D_{2}$	4 mm	74,714	11,632	11,006	15,800	6530	5988	0.6	—	4.94	—
	5 mm	39,643	7021	13,866	8064	3878	7481	0.631	5.17%	4.96	0.40%
	7 mm	16,280	4097	3953	3105	2216	2001	0.643	7.17%	4.82	2.43%
	8 mm	12,667	3373	3600	2400	1807	1809	0.546	9.00%	4.72	4.45%
$D_{4}$	4 mm	74,714	12,927	13,774	15,800	7220	7543	0.142	—	7.677	—
	5 mm	39,643	7455	8665	8064	4075	4639	0.138	0.35%	7.65	2.82%
	7 mm	16,280	4130	4150	3105	2222	2102	0.128	0.69%	7.624	9.86%
	8 mm	NA	NA	NA	NA	NA	NA	NA	NA	NA	NA
$D_{6}$	4 mm	74,714	17,399	25,057	15,800	9871	13,753	0.149	—	11.08	—
	5 mm	39,643	10,766	12,905	8064	6027	6852	0.1433	3.83%	10.937	1.29%
	7 mm	16,280	5822	4829	3105	3197	2410	0.136	8.72%	10.775	2.75%
	8 mm	12,667	4520	4170	2400	2447	2048	0.131	12.08%	10.71	3.34%
$D_{7}$	4 mm	74,714	13,678	10,259	15,800	7696	5557	0.111	—	9.366	—
	5 mm	39,643	8195	6454	8064	4532	3386	0.109	1.80%	9.399	0.35%
	7 mm	16,280	4809	4186	3105	2599	2129	0.107	3.60%	9.212	1.64%
	8 mm	12,667	4030	3925	2400	2187	1973	0.104	6.31%	9.302	0.68%
$D_{8}$	4 mm	74,714	8935	8789	15,800	4957	4649	0.167	—	11.864	—
	5 mm	39,643	6424	6652	8064	3548	3417	0.165	0.51%	11.925	1.20%
	7 mm	16,280	3697	3961	3105	1977	1919	0.18	9.44%	10.744	7.78%
	8 mm	12,667	3131	7025	2400	1677	3538	0.129	2.92%	12.21	22.75%
$D_{9}$	4 mm	74,714	13,678	26,909	15,800	7696	14,950	0.09	—	8.85	—
	5 mm	39,643	8195	18,401	8064	4532	10,103	0.087	3.33%	8.84	0.11%
	7 mm	16,280	4809	19,811	3105	2599	10,988	0.09	0%	8.76	1.02%
	8 mm	12,667	4030	4802	2400	2187	2475	0.091	1.11%	8.755	1.07%

Abbreviations: MS—Mesh Size (mm). NG—Number of nodes in the ground; NF—Number of nodes in the foot; NI—Number of nodes in the insole. EG—Number of elements in the ground; EF—Number of elements in the foot; EI—Number of elements in the insole. PPP—Peak plantar pressure (MPa); ΔPPP—Percentage difference in PP relative to fine mesh. TD—Total deformation (mm); ΔTD—Percentage difference in TD relative to fine mesh. Note: 4 mm of mesh served as the fine-mesh reference.
